# Antimicrobial use in an Indonesian community cohort 0-18 months of age

**DOI:** 10.1371/journal.pone.0219097

**Published:** 2019-08-05

**Authors:** Jarir At Thobari, Cahya Dewi Satria, Yohanes Ridora, Emma Watts, Amanda Handley, Samad Samad, Novilia S. Bachtiar, Julie E. Bines, Yati Soenarto, Jim P. Buttery

**Affiliations:** 1 Pediatric Research Office, Department of Pediatrics, Faculty of Medicine, Public Health and Nursing, Universitas Gadjah Mada, Yogyakarta, Special Region of Yogyakarta, Indonesia; 2 Department of Pharmacology and Therapy, Faculty of Medicine, Public Health and Nursing, Universitas Gadjah Mada, Yogyakarta, Special Region of Yogyakarta, Indonesia; 3 RV3 Rotavirus Vaccine Program, Murdoch Children’s Research Institute, Parkville, Victoria, Australia; 4 Medicines Development for Global Health, Melbourne, Victoria, Australia; 5 Soeradji Tirtonegoro General Hospital, Klaten, Central Java, Indonesia; 6 PT Bio Farma, Bandung, West Java, Indonesia; 7 Department of Pediatrics, University of Melbourne, Melbourne, Victoria, Australia; 8 Department of Gastroenterology and Clinical Nutrition, Royal Children’s Hospital Melbourne, Parkville, Victoria, Australia; 9 Department of Paediatrics, Monash University, Clayton, Victoria, Australia; 10 School of Public Health and Preventive Medicine, Monash University, Clayton, Victoria, Australia; 11 Department of Infection and Immunity, Monash Children’s Hospital, Clayton, Victoria, Australia; Federal University of Sergipe, BRAZIL

## Abstract

**Background:**

Antimicrobial resistance has become a global health emergency and is contributed to by inappropriate antibiotic use in community clinical settings. The aim of this study was to evaluate the antimicrobial use pattern in infants from birth until 18 months of age in Indonesia.

**Methods:**

A post-hoc analysis was conducted in 1621 participants from the RV3BB Phase IIb trial conducted in Indonesia from January 2013 through July 2016. Any health events were documented in the trial as adverse events. Concomitant medication surveillance recorded all medications, including antibiotics during the 18 months of follow-up. Information included the frequency, duration of usage, formulation, classes, and their indications, including prophylactic antibiotic and perinatal use.

**Results:**

Of 1621 participants, 551 (33.99%) received at least one antibiotic for treatment of infections during the 18 months observation period. Additionally, during the perinatal period, prophylactic antibiotics were used in 1244 (76.74%) participants and antibiotics consumed in 235 mothers of participants (14.50%). A total of 956 antibiotic consumptions were recorded for 18 months follow up, 67 (7.01%) as part of antimicrobial combinations. The average duration of antibiotic course was 4.92 days. Penicillin and sulfonamides were the most common antibiotic classes consumed (38.81% and 24.48%, respectively).

**Conclusions:**

Despite the low community consumption rate, the overuse of antibiotic in URTIs and non-bloody diarrhea in our setting represents a major opportunity for antimicrobial stewardship, particularly in early life.

## Introduction

Antibiotics are widely used among adults and children in the community. In children, antibiotics are considered a potential lifesaving treatment for bacterial infections. International guidelines for Integrated Management of Childhood Illness (IMCI) recommended antibiotic use to treat dysentery and acute respiratory tract infection [1]. However, in both developed and developing settings, antibiotics are often used inappropriately, such as for non-specific respiratory illnesses and non-bloody diarrhea [2,3].

Inappropriate use of antibiotics can also lead to antimicrobial resistance which is recognized as a serious global health threat [4]. In infancy, antibiotic use is associated with a long-term decrease in the diversity of microbiota, and an increase in inflammatory bowel disease, atopic diseases, and obesity [5–7]. Antimicrobial resistance has become a global health emergency with emergence of antibiotic resistance outpacing the development of new antimicrobials.

As the fourth most populated country in the world, Indonesia’s population in 2018 is 266 million people, with 29% under 15 years of age. Among the medications given to children, antibiotics are the most frequently prescribed therapy [5,8]. In high-income countries, high rates of antibiotic utilization in the community, hospitals, and agriculture have played an important role in selection pressure that has maintained resistant strains, resulting in clinicians choosing wider-spectrum and more expensive antibiotics [9]. Meanwhile, in LMICs, the use of antibiotics is growing with the rise in incomes, high rates of hospitalization and hospital-acquired infections [10]. Key to understanding ways to decrease antimicrobial resistance is by understanding how antimicrobials are used in the community, especially in young children who are typically the most frequent consumers of antibacterial agents.

Studies of antibiotic use have been performed in diverse health-care settings and in community-based surveys. However, such studies are still very limited in Indonesia. In Indonesia, antibiotics require a prescription to be dispensed, however, previous studies have shown high rates of pharmacy provision of antibiotics without a prescription [11]. The RV3-BB phase IIb trial assessed the safety and efficacy of the RV3-BB vaccine against rotavirus gastroenteritis. As part of active participant follow-up, this included surveillance of concomitant medication consumed by the participants. The RV3 concomitant medication data represents a unique opportunity to explore routine antimicrobial consumption in 1621 children in Indonesia followed from pregnancy to 18 months of age. Thus, the aim of this study is to describe the pattern of antibiotics use and the indications of the administration of antibiotics in subjects of RV3-bb phase IIb trial. This may help Indonesian and international authorities in developing strategies for community-based antimicrobial stewardship programs.

## Methods

### Study design

The secondary data from a phase IIb randomized, double-blinded, controlled trial for RV3-BB rotavirus vaccine (RV3-BB phase IIb trial) was used in this study (Australian New Zealand Clinical Trials Registry number ACTRN12612001282875; the protocol is available at NEJM.org). The complete study design of RV3-BB phase IIb trial has been described previously [12] and is summarized here. The primary objective of RV3-BB phase IIb trial was to evaluate the efficacy of the RV3-BB vaccine against severe rotavirus gastroenteritis compared to placebo, in children up to 18 months of age. The participants were randomized into three groups: neonatal and infant active dosing, and placebo. Each participant received four oral doses of vaccines or placebo according to their trial-group assignment. Neonatal and infant-schedule vaccine group received 3 doses of RV3-BB vaccines and 1 dose of placebo. In neonatal-shedule vaccine group, RV3-BB vaccines were administered at dose 1, 2, and 3, and followed by placebo at dose 4. In infant-schedule vaccine group, placebo was administered at dose 1, and followed by RV3-BB vaccines at dose 2, 3, and 4. Dose 1 was administered at 0 to 5 days of age, dose 2 at 8 to 10 weeks of age, dose 3 at 14 to 16 weeks of age, and dose 4 at 18 to 20 weeks of age. Participants were followed up until 18 months of age. Prospective adverse event surveillance included scheduled visits and weekly telephone calls by study personnel. Concomitant medication surveillance recorded details of every medication consumed by the participants during the 18 months of follow up.

### Participants, randomization, and blinding

**Fig 1:**
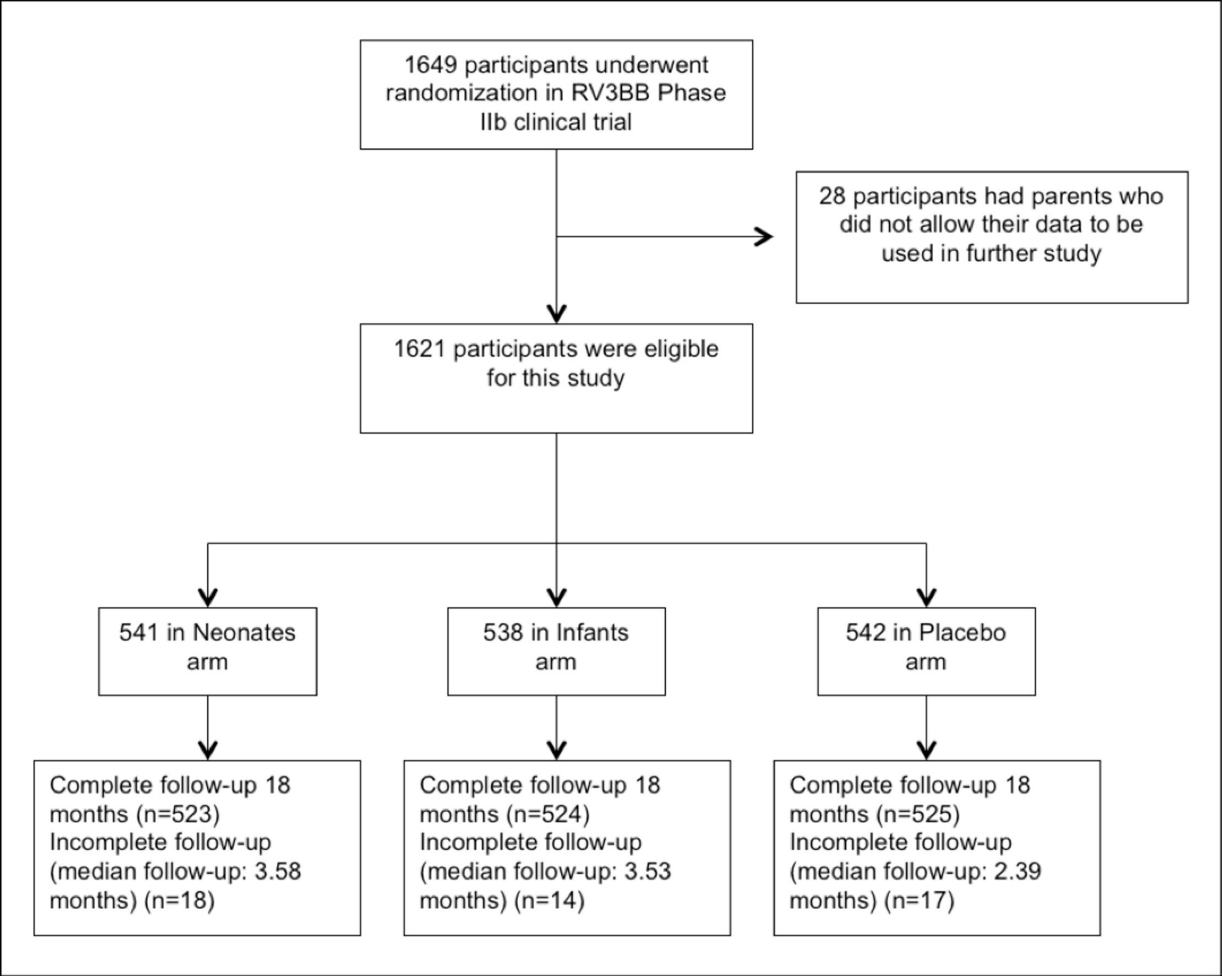
Flow diagram of participants follow-up.

RV3-BB phase IIb trial was conducted in two districts in Yogyakarta and Central Java province in Indonesia from January 2013 through July 2016, with a total of 1649 participants were enrolled and assigned to one of the three trial groups. Of 1649 participants, 1621 consented that their data will be used in the future research, including in this post-hoc analysis (see Fig 1). We included 49 participants who were not completely followed until 18 months in the analysis because some of these participants used at least one antibiotic before being lost to follow-up. This information was considered valuable in this study, and since the proportion of the lost to follow-up participants was only 3%, it might not lead to bias.

The study sites were 23 Primary Health Care (PHCs) and 2 hospitals in Sleman district (Yogyakarta province) as urban area and Klaten district (Central Java province) as rural area. After initial provisional maternal written informed consent in pregnancy, healthy neonates were enrolled in this study in less than 6 days of age. The inclusion criteria were children who were healthy, born full-term, weighed between 2500 and 4000 grams. Eligible participants were randomly assigned to one of the three groups with a 1:1:1 ratio. Randomization was completed based on a computer-generated code with a block size of 6, with stratification according to province. All investigators, trial monitors, data managers, statisticians, other trial staffs, and participants’ families remained blind to the trial-group assignments until the completion of the trial. At the central pharmacy in each province, pharmacist who was aware of the trial-group assignments drew the doses of RV3-BB vaccine or placebo for dispensing [12].

### Sample size

The detailed explanation on sample size calculation has been described previously [12]. In brief, to preserve the trial with 80% power to reject the null hypothesis of no difference between the combined vaccine group and the placebo group, our calculation generated an enrollment target of 549 participants in each of the three trial groups (assuming that 3% of the placebo group would have a severe rotavirus gastroenteritis, the true efficacy of the vaccine was 60%, at a one-sided apha level of 0.1). The allowance for nonadherence rate to the trial regimen with this sample size was 10%.

### Antibiotics

Antibiotics as a part of concomitant medication were recorded from perinatal exposure from antibiotics given to the mother during the labour up to 18 months of age. Antibiotic use by participants’ mother during perinatal period in this study were recorded from 30 days to several hours prior to labour. After gave birth, during weekly follow up phone calls, research assistants would ask the mothers about the condition of the participants, including when there was any medication given to the participants from previous contact. Study midwives visited participants at their home monthly and monitored medications given to participants during the study by asking the mothers. If the participants were ill and visited *Puskesmas* (primary health care facilities, PHC), medications were also documented in medicals records. If the participants were hospitalised, medications given to them were extracted from their medical records. All the follow up data were inputted into an electronic case report form.

From the concomitant medications data, the information on antibiotics used by the study participants were extracted, both prescribed and non-prescribed antibiotics. Collected Information on antibiotics included antimicrobial type, class, dose, formulation such as oral (puyer/pulveres, syrup), parenteral or topical, mode of administration, duration of course, and their indications. *Puyer*, derived from “powder” in Dutch, contains medicines all ground together and has been commonly used for a long time in Indonesia, including for antibiotics [13]. Information about antibiotic combination therapy was also generated. Combination therapy was defined as antibiotic consumption containing more than one type of antimicrobial (counted as one consumption event). Despite the fact that trimethoprim and sulfamethoxazole were two different antibiotics, these substances were almost always given together as a treatment for children (according to the national and international guidelines recommendations of childhood illness management). Therefore, for the purpose of interpretation, we considered cotrimoxazole as a single antibiotic therapy.

### Episodes of adverse events recorded

Adverse event (AE) episodes were defined as all illnesses or symptoms occurred during the 18 months observation period following the first dose of RV3 vaccine or placebo was given at day 0–6 of life. Adverse events were classified using Medical Dictionary for Regulatory Activities (MedDRA). The final classification of morbidities was narrowed down to 13 disorders mainly according to their affected organ systems.

### Ethical considerations

This study received the ethical approval from Medical and Health Research Ethics Committee of Faculty of Medicine Universitas Gadjah Mada–Dr Sardjito General Hospital. Written informed consent from the parents or guardians of every child were already obtained during the RV3-BB phase IIb trial. The subjects who were included in this study were those who agreed that their data will be used for further studies.

### Statistical analysis

All statistical calculations were conducted using SPSS version 23. Results were presented as mean, median, frequency and percentages for descriptive data. Bivariate chi-square test and binary logistic regression of association was carried out to explore the relationship between gender, vaccination group and the number of AE episodes with antibiotic use. The independent t-test was conducted to compare the differences in antibiotic course duration per participant between males and females. One-way ANOVA was also performed to explore the effect of vaccination to the antibiotic course duration per participant.

## Results

### Baseline characteristics

551 of 1621 (33.99%) participants received at least one therapeutic antibiotic during the 18-month observation period. Baseline characteristics of the study participants can be seen at Table 1. Male (p = 0.015) and the number of AE episodes (p = 0.00) were significantly associated with the incidence of antibiotic use. There was no difference in antibiotic use in participants that received vaccine or placebo (neonatal-schedule p = 0.19; infant-schedule p = 0.78). In addition, single topical-prophylactic agents, including chloramphenicol, gentamycin, and tetracycline, were used in 76.74% (n = 1244/1621) participants directly after birth to prevent ophthalmia neonatorum (ON).

**Table 1:** Baseline characteristics of the study participants with antibiotic treatment.

Characteristics	All (n = 1621)	With antibiotic use (n = 551)	Without antibiotic use (n = 1070) *	P-value	OR (95% CI)
Gender (%)					
Male	844 (52.07)	310 (56.26)	534 (49.91)	0.015	1.29 (1.05–1.59)
Female	777 (47.93)	241 (43.74)	536 (50.09)	Ref	Ref
Vaccination group (%)					
Neonates	541 (33.37)	196 (35.57)	343 (32.06)	0.19	1.18 (0.92–1.52)
Infants	538 (33.19)	179 (32.49)	359 (33.55)	0.78	1.04 (0.80–1.34)
Placebo	542 (33.44)	176 (31.94)	366 (34.21)	Ref	Ref
Episodes of AE recorded					
Mean ± SD	5 ± 0.74	6.47 ± 1.89	4.34 ± 2.13	0.00	
Median (p25-p75)	5 (3–8)	7 (4–10)	4 (2–7)		
Min-max	1–30	1–30	0–29		

* Including those receiving concomitant medications other than antibiotics

### Antibiotic use pattern

A total of 956 antibiotic courses were recorded in 551 infants during a 18-month observation period, with an average of 1.74 antibiotic use per infant (Table 2). The mean duration of antibiotic use per child was 4.92 (±1.86) days. Neither gender (p = 0.70) nor vaccination group (p = 0.98) were associated with the duration of antibiotic course. Antimicrobial combinations comprised only seven percent of the uses.

**Table 2:** The pattern of antibiotic use as treatment.

Pattern indicator (n = 956)	Results	P-value
**Duration of antibiotic use (days) (mean ± SD)**	4.92 ± 1.86	
Gender		0.70
Male	4.87 ± 1.85	
Female	4.98 ± 1.88	
Vaccination group		0.98
Neonates	4.94 ± 1.83	
Infants	4.89 ± 1.79	
Placebo	4.93 ± 1.97	
**Combination of antibiotics [n(%)]**		
Single antimicrobial	889 (92.99)	
Double-antimicrobial	66 (6.90)	
Triple-antimicrobial	1 (0.10)	
**Antibiotic formulation [n(%)]**		
Oral (syrup/drop/pulveres)	615 (64.33)	
Topical (skin/ear/eye preparation)	249 (26.05)	
Parenteral (intravenous)	89 (9.31)	
**Antibiotic usage for hospitalized/** **non-hosptialized adverse event (%)**		
Non-hospitalized	817 (85.46)	
Hospitalized	139 (14.54)	

Oral antibiotic formulations were used in 64.33% of all antibiotic consumptions, which consisted of syrups, pulveres/puyer, and unknown oral formulations. All parenteral antibiotics were administered intravenously. Most of antibiotic use was for outpatient therapy.

### Classes of antibiotic and indications

Penicillin was the most common antibiotic class used for treatment (n = 371/956; 38.81%), with 68.46% of penicillin utilization aimed to treat respiratory system disorders. Although mostly consumed as single antimicrobial (322/371), penicillin was the most frequent antibiotic class used in combination therapy (49/67), especially with aminoglycosides (22/49). The penicillin-aminoglycosides combination therapy was preferred in the pneumonia (6/22) and neonatal sepsis (5/22). The second largest antibiotic class was sulfonamides (n = 234/956; 24.48%), overwhelmingly as cotrimoxazole and mostly indicated for treating gastrointestinal disorders (50.85%). The antibiotics and their usage indications are summarized in Table 3. Respiratory and gastrointestinal disorders were the most common morbidities recorded during our observation period, with episodes treated by antibiotics was 18.06% and 6.98%, respectively. Adverse event episodes recorded during the study period are summarized in Table 4.

**Table 3:** Classes of antibiotics and indication of treatment.

Classes of antibiotic	N(%)	Single / combination	N (%)	Indication of use (five most common) (N (%))
**Penicillin**	371 (38.81)	Single	322 (86.79)	Respiratory system disorders [254 (68.46)]; Skin disorders [32 (8.62)]; Unspecified pyrexia [17 (4.58)]; Gastrointestinal disorders [15 (4.04)]
Amoxicillin	322 (33.68)	Combination	49 (13.21)	
Ampicillin	48 (5.02)			
Benzyl penicillin	1 (0.10)			
**Sulfonamides**	234 (24.48)	Single	229 (97.86)	Gastrointestinal disorders [119 (50.85)]; Respiratory system disorders [96 (41.02)]; Unspecified pyrexia [9 (3.84)]; Metabolism and nutrition disorders [3 (1.28)]
Cotrimoxazole	231 (24.16)	Combination	5 (2.14)	
Sulfacetamide	2 (0.21)			
Silver sulfadiazine	1 (0.10)			
**Amphenicols**	109 (11.40)	Single	98 (89.90)	Ocular disorders [66 (60.55)]; Skin disorders [15 (13.76)]; Respiratory system disorders [12 (11.01)]; Gastrointestinal disorders [5 (4.59)]
Chloramphenicol	108 (11.30)	Combination	11 (10.19)	
Thiamphenicol	1 (0.10)			
**Aminoglycosides**	124 (12.97)	Single	90 (72.58)	Skin disorders [78 (62.90)]; Ocular disorders [14 (11.29)]; Respiratory system disorders [12 (9.67)]; Other infections [12 (9.67)]
Gentamicin	93 (9.73)	Combination	34 (27.42)	
Amikacin	7 (0.73)			
Neomycin	20 (2.09)			
Tobramycin	3 (0.31)			
Netilmicin	1 (0.10)			
**Third-generation cephalosporin**	42 (4.39)	Single	32 (76.19)	Gastrointestinal disorders [14 (33.33)]; Respiratory system disorders [13 (30.95)]; Genitourinary system disorders [8 (19.04)]; Central-peripheral nervous system [3 (7.14)]
Cefixime	13 (1.36)	Combination	10 (23.81)	
Cefotaxime	19 (1.99)			
Ceftazidime	4 (0.42)			
Ceftriaxone	6 (0.63)			
**Tetracycline**	31 (3.24)	Single	28 (90.32)	Ocular disorders [18 (58.06)]; Skin disorders [11 (35.48)]; Gastrointestinal disorders [1 (3.22)]; Ear disorders [1 (3.22)]
Oxytetracycline	29 (3.03)	Combination	3 (9.68)	
Tetracycline	2 (0.22)			
**Other antibacterial**	113 (11.82)	Single	90 (79.65)	See S1 Table
		Combination	23 (20.35)	

**Table 4:** Adverse event episodes recorded during the study period.

No	Adverse event	Child (N (%))	Total episodes of AE (N (%))	Episodes of AE treated by antibiotic N (%)
1	Ocular disorders	88 (5.80)	95 (0.98)	77 (81.05)
2	Lymphatic system disorders	3 (0.20)	3 (0.03)	2 (66.67)
3	Ear disorders	23 (1.52)	25 (0.26)	15 (60)
4	Other infection disorders	54 (3.56)	59 (0.61)	25 (42.37)
5	Genitourinary disorders	39 (2.57)	43 (0.44)	13 (30.23)
6	Skin disorders	408 (26.88)	550 (5.66)	128 (23.27)
7	Respiratory system disorders*	995 (65.55)	1854 (19.10)	335 (18.06)
8	Other viral infections	42 (2.77)	42 (0.43)	6 (14.29)
9	Metabolism and nutrition disorders	29 (1.91)	30 (0.31)	3 (10)
10	Central-peripheral nervous system	55 (3.62)	60 (0.62)	5 (8.33)
11	Gastrointestinal disorders**	985 (64.89)	2335 (24.05)	163 (6.98)
12	Unspecified pyrexia	935 (61.59)	1587 (16.35)	29 (1.83)
13	Other disorders	942 (62.06)	3026 (31.17)	6 (0.20)
	Total	1518 (100)	9709 (100)	807 (8.31)

*292 of 335 (84.78%) respiratory illnesses treated with antimicrobial belonged to URTIs

**122 of 163 (74.85%) gastrointestinal disorders treated with antimicrobial were non-bloody diarrhea

### Antibiotic prophylaxis and perinatal use

Aside of therapeutic indications, prophylactic usages of antimicrobials were also observed during the study. Single topical-prophylactic agents, including chloramphenicol, gentamycin, and tetracycline, were used in 76.74% (n = 1244/1621) participants directly after birth to prevent ophthalmia neonatorum (ON). None of the participants received more than one type of antibiotic prophylaxis during the study.

There were 235 participants’ mothers (14.50%) who received antibiotics during the perinatal period with start dates ranged between 30 days to several hours prior to labour. Ceftriaxone, cefotaxime, cefadroxil, amoxicillin, and metronidazole comprised 98.30% of all perinatal antibiotic use in this study (51.06%, 23.40%, 11.91%, 8.51, and 3.40%, respectively). None of the participants’ mother used more than one antibiotic during the observation period. All the prophylactic and perinatal use of antibiotics can be seen at Table 5.

**Table 5:** List of antibiotic prophylaxis and perinatal use.

Prophylaxis use (%)* n = 1244		Perinatal use (%) n = 235	
Antibiotic type	N(%)	Antibiotic type	N(%)
Chloramphenicol	519 (41.72)	Ceftriaxone	120 (51.06)
Gentamycin	406 (32.64)	Cefotaxime	55 (23.40)
Tetracycline	319 (25.64)	Cefadroxil	28 (11.91)
		Amoxicillin	20 (8.51)
		Metronidazole	8 (3.40)
		Other antibiotics	4 (1.70)

*All in topical eye preparations

## Discussion

### Antibiotic use pattern

One in three participants (33.99%) had at least one antibiotic used for treatment of infections within the 18 months observation period. This number was surprisingly low and may reflect a strength, especially since the study captured both prescribed and non-prescribed antibiotic use. Some studies in other developing countries showed much higher percentages of antibiotic consumption among children, including: India, Sudan, and Nigeria (79%, 81.3% and 71.1%, respectively) [14–16]. This is encouraging and may also reflect the success of antimicrobial stewardship implementation in Yogyakarta and Central Java province in Indonesia. Moreover, a systematic review on impact of antibiotic stewardship programme (ASPs) in Asia also showed a reduction of antibiotic consumption in 91% of studies and cost savings in 100% studies due to ASPs in hospital and clinic settings [17]. It had been suggested that the increase of antibiotic use in LMICs was correlated with rising incomes, high-rates of hospitalization, and high-prevalence of hospital infections [9]. Unfortunately, in health-care settings, the transmission of a resistant bacteria can be prompt and have severe outcomes for vulnerable hosts [10]. Therefore the rational use of antibiotics is a priority.

Gender was significantly associated with antibiotic use in this study, with more male used antibiotic than female. This result was similar with other previous studies in which the male predominance in antibiotic prescription rate might reflect the difference of infectious disease burden between boys and girls in younger age [2,8]. Several studies suggested a reduction of antibiotic uses among population receiving vaccinations, particularly in Group B streptococcal (GBS), respiratory syncytial virus (RSV), influenza, *Haemophilus influenzae* type b (Hib), and pneumococcal conjugate vaccine (PCV). This may be due to the directly-diminished primary pathogens causing infections and also through the declining of febrile illnesses which often led to antibiotic prescriptions [18–20]. Report of rotavirus vaccination on reducing the overall antibiotic use in children was still rare compared to other existing vaccines. In this study, no significant association between rotavirus vaccination and the incidence and duration of antibiotic use. This partly reflects that rotavirus vaccine might not reduce inappropriate antibiotic use which caused by bacterial or other viral infections. The main objective of rotavirus vaccine development was to reduce the morbidity and mortality of rotavirus infection, but the administration of other existing vaccines should be done to prevent further inappropriate antibiotic use [18,20].

The mean duration of antibiotic courses was 4.92 (±1.86) days per participants. This correlates well with a similar study of antibiotic use patterns in eight countries in South America, Sub-Saharan Africa and Asia [2]. Several factors should be considered in determining the duration of antibiotic use to decrease further antimicrobial resistance. WHO recommends a 3 day antibiotic course for non-severe pneumonia diagnosed in low-resource settings [21]. However, even a short treatment course of antibiotic will be the worst and potentially harmful strategy when it is not needed, as in viral respiratory infections [22].

### Antibiotic formulations

Of all antibiotics used in this study, two thirds contained oral antibiotic formulations. This is reasonable as oral is the preferred route for patients of all ages in terms of convenience [23]. Syrup and pulveres were the oral formulations used by our participants, since neonates and infants appeared to have difficulty swallowing oral solid formulations [24]. A study concerning on the acceptability of different oral formulations among children between 1–4 years of age also showed that small tablet and syrup were the most preferred formulations by parents and children, although suspension and pulveres were also well accepted [25]. Pulveres is a commonly used drug formulation in Indonesia due to the lower price and its convenience to be combined with other drugs, yet also increase the probability of drug interactions [26,27].

### Antibiotic classes and indications

Among the numerous kinds of antibiotic recorded, penicillin was the most frequent class used, followed by sulfonamides. The most common reason (68.46%) for using penicillin was respiratory system disorders, while sulfonamides were mostly (50.85%) aimed for gastrointestinal disorders. Similarly, studies showed that penicillin was the most frequent antibiotic class used in children [28], especially to treat respiratory illnesses [2]. This might be due to streptococcal species being most common bacteria causing RTIs, thus penicillin are the group of choice to treat bacterial RTIs [29]. Penicillin was also the most common antibiotic used as combination therapy in this study (49/67), mostly with aminoglycosides which were mainly indicated for neonatal sepsis and pneumonia. This was in line with international guideline as the combination of gentamicin and ampicillin or benzylpenicillin was recommended for infants with a possible serious bacterial infection [30].

However, among adverse events that were treated by antibiotic, URTIs comprised 84.78% of the respiratory system disorders, whereas non-bloody diarrhea comprised 74.85% of the gastrointestinal disorders. These results were contrary to international guidelines for the treatment of childhood illnesses which recommended that antibiotic should not be routinely prescribed for non-bloody diarrhea and URTIs [1, 2, 31]. Most URTIs are caused by a viral infection which do not need antibiotics for the treatment. The administration of antibiotic therapy in children with non-streptococcal pharyngitis and common cold has not demonstrated benefit in a recent study by Lindell et al. (2011) [32].

Chloramphenicol utilization comprised 11.30% of all antibiotics and mostly aimed for ocular disorders. Since the dosing is not weight-adjusted, ocular treatment administration in children should be well-observed as they are at greater risk of systemic side effects, and drug metabolism is reduced in the immature blood-brain barrier [33]. Macrolides and cephalosporin were not frequently used as the other antibiotic classes in this study. This might be due to the socioeconomic factor as previously suggested by another study [2].

### Antibiotic prophylaxis and perinatal use

Topical ocular antibiotics were widely used among participants as the national guideline in essential newborn care requires all the newborns to receive these antimicrobials to prevent ophthalmia neonatorum (ON) [34,35]. About one fourth of the newborns in our setting did not get ocular prophylactic antibiotic, indicating that the adherence to the guideline was still below expectation. This implies the need for further evaluation of and education for childbirth helper regarding the newborn care guideline compliance. However, the regulation of topical antibiotic prophylaxis for ON is widely varied across the countries. For example, preventive treatment for ON was not routinely used in Britain, Denmark, Sweden and Norway, while in the USA, Israel, Mexico, and Austria, prophylactic treatments for ON were recommended [36–40]. In countries where ON topical preventive treatment of newborn was routinely applied, a variety of topical agents were used, such as 1% silver nitrate, 1% tetracycline, macrolides (0.5% erythromycin or azithromycin), aminoglycosides (gentamicin and tobramycin), chloramphenicol or povidone-iodine [37]. In Indonesia, 1% chloramphenicol ointment was commonly used as preventive agent against ON [41]. Although its systemic side effects in aplastic anemia have been reported before, the occurrence of aplastic anemia among ocular use of chloramphenicol is extremely low [42]. In countries where universal prophylaxis for ON were abandoned, prenatal screening and treatment of sexually transmitted diseases have decreased the incidence of ON [36].

Almost 1 in 6 participants’ mother consumed antibiotic during the perinatal period. Recent study suggested an association between intrapartum ampicillin with the emergence of ampicillin-resistant *E*.*coli* infections at birth [43,44]. Moreover, several studies have showed an influence of maternal antibiotic therapy upon the development the infant microbiome. Inappropriate use of antibiotic during pregnancy has been suggested to affect the maturation of the child’s immune system which could lead to many conditions, including allergic diseases [45–47]. Therefore, antibiotic prescribing during the perinatal period should be for clear indications.

### Rational use of antibiotics

Considering a large sample size used in our study, the results might represent the antibiotic utilization in wider population of young children in Yogyakarta and Central Java provinces. However, different result might occur in different area in Indonesia. Many aspects could affect clinician’s decision making for prescribing antibiotics in children. Misinterpretation of parental concerns over their ill children and seeking of additional information, and lacking of time during consultation are the problems most commonly encountered [48]. Thus, better communication between primary care physicians and parents [49,50], improved parents’ understanding of self-limiting diseases and the appropriate therapy [51,52], and sufficient time in consultation might reduce unnecessary antibiotic prescriptions [48]. Another challenging situation faced by LMICs countries on giving appropriate therapy is that diagnosis is difficult in low-resource settings and often rely only on clinical symptoms and provided algorithms [53].

In Indonesia, although there are policies that only allow antibiotic purchase by prescriptions, antibiotics are still relatively easily obtained without a prescription. These behaviors may contribute to evolving antimicrobial resistance. Antimicrobial resistance is a complex global health issue, and no simple strategy will be enough to conquer it, because of that, the development and implementation of holistic strategies to restrict the emersion and spread of antimicrobial resistance are vital [54].

## Conclusion

Despite the low community consumption rate, the overuse of antibiotic in URTIs and non-bloody diarrhea among infants in this study indicated inappropriate antimicrobial use and further opportunities for education. Moreover, different classes of antibiotic were inappropriately used and may risk development of antimicrobial resistance. Training on rational use of antibiotic is needed to improve prescriptions behavior of practitioners in clinical settings.

## Supporting information

S1 Checklist Consort checklist of post-hoc analysis of RV3-BB phase IIb trial. (DOCX)

S1 Table Classes of antibiotics used and reason for prescribing. (DOCX)

S1 Dataset Antibiotic use in children aged 0–18 months. (XLSX)
