# Supplementary material for: Antimicrobial use in an Indonesian community cohort 0-18 months of age
Source: PLoS One. 2019 Aug 5;14(8):e0219097. doi: 10.1371/journal.pone.0219097 (PMC6681970; doi:10.1371/journal.pone.0219097)
Supplement: S1 Table — (DOCX) [file pone.0219097.s002.docx]

| **S1 Table. Classes of antibiotics used and reason for prescribing** | | | | |
| --- | --- | --- | --- | --- |
| Classes of antibiotic | N(%) | Single antimicrobial/  combination | N(%) | Indication for prescribing (%) |
| **Penicillin** | 371 (38.81) | Single antimicrobial | 322 (86.79) | Respiratory system disorders (68.46); Skin disorders (8.62); Unspecified pyrexia (4.58); Gastrointestinal disorders (4.04) |
| Amoxicillin | 322 (33.68) | Combination | 49 (13.21) |  |
| Ampicillin | 48 (5.02) |  |  |  |
| Benzyl penicillin | 1 (0.10) |  |  |  |
|  |  |  |  |  |
| **Sulfonamides** | 234 (24.48) | Single antimicrobial | 229 (97.86) | Gastrointestinal disorders (50.85); Respiratory system disorders (41.02); Unspecified pyrexia (3.84); Metabolism and nutrition disorders (1.28) |
| Cotrimoxazole | 231 (24.16) | Combination | 5 (2.14) |  |
| Sulfacetamide | 2 (0.21) |  |  |  |
| Silver sulfadizine | 1 (0.10) |  |  |  |
|  |  |  |  |  |
| **Amphenicols** | 109 (11.40) | Single antimicrobial | 97 (89.90) | Ocular disorder (60.55); Skin disorders (13.76); Respiratory system disorders (11.01); Gastrointestinal disorders (4.59) |
| Chloramphenicol | 108 (11.30) | Combination | 11 (10.19) |  |
| Thiamphenicol | 1 (0.10) |  |  |  |
|  |  |  |  |  |
|  |  |  |  |  |
| **Aminoglycosides** | 124 (12.97) | Single antimicrobial | 90 (72.58) | Skin disorders (62.90); Ocular disorders (11.29); Respiratory system disorders (9.67); Other infections (9.67) |
| Gentamicin | 93 (9.73) | Combination | 34 (27.42) |  |
| Amikacin | 7 (0.73) |  |  |  |
| Neomycin | 20 (2.09) |  |  |  |
| Tobramycin | 3 (0.31) |  |  |  |
| Netilmycin | 1 (0.10) |  |  |  |
|  |  |  |  |  |
| **Third-generation cephalosporin** | 42 (4.39) | Single antimicrobial | 32 (76.19) | Respiratory system disorders (30.95); Gastrointestinal disorders (33.33); Genitourinary system disorders (19.04); Central-peripheral nervous system (7.14) |
| Cefixime | 13 (1.36) | Combination | 10 (23.81) |  |
| Cefotaxime | 19 (1.99) |  |  |  |
| Ceftazidime | 4 (0.42) |  |  |  |
| Ceftriaxone | 6 (0.63) |  |  |  |
|  |  |  |  |  |
| **Tetracycline** | 31 (3.24) | Single antimicrobial | 28 (90.32) | Ocular disorders (58.06); Skin disorders (35.48); Gastrointestinal disorders (3.22); Ear disorders (3.22) |
| Oxytetracycline | 29 (3.03) | Combination | 3 (9.68) |  |
| Tetracycline | 2 (0.22) |  |  |  |
|  |  |  |  |  |
|  |  |  |  |  |
| **First-generation cephalosporin** | 20 (2.10) | Single antimicrobial | 19 (95) | Gastrointestinal disorders (15); Genitourinary system disorders (20); Respiratory system disorders (15); Unspecified pyrexia (15) |
| Cefadroxil | 20 (2.10) | Combination | 1 (5) |  |
|  |  |  |  |  |
|  |  |  |  |  |
|  |  |  |  |  |
| **Macrolides** | 14 (1.46) | Single antimicrobial | 9 (64.28) | Skin disorders (42.85); Respiratory system disorders (35.71); Other infections disorders (14.28); Central-peripheral nervous system disorders (7.14) |
| Erythromycin | 13 (1.36) | Combination | 5 (35.71) |  |
| Azithromycin | 1 (0.10) |  |  |  |
|  |  |  |  |  |
|  |  |  |  |  |
| **Fluoroquinolones** | 7 (0.73) | Single antimicrobial | 7 (100) | Gastrointestinal disorders (71.42); Ear disorders; (14.28); Other infections disorders (14.28) |
| Ciprofloxacin | 6 (0.63) | Combination | 0 (0) |  |
| Ofloxacin | 1 (0.10) |  |  |  |
|  |  |  |  |  |
| **Carbapenems** | 2 (0.21) | Single antimicrobial | 2 (100) | Gastrointestinal disorders (100) |
| Imipenem | 2 (0.21) | Combination | 0 (0) |  |
|  |  |  |  |  |
| **Fourth-generation cephalosporin** | 1 (0.10) | Single antimicrobial | 1 (100) | Other infections disease (100) |
| Cefepime | 1 (0.10) | Combination | 0 (0) |  |
|  |  |  |  |  |
| **Second-generation cephalosporin** | 2 (0.21) | Single antimicrobial | 1 (50) | Gastrointestinal disorders (100) |
| Cefuroxime | 2 (0.21) | Combination | 1 (50) |  |
|  |  |  |  |  |
| **Lincosamides** | 1 (0.10) | Single antimicrobial | 1 (100) | Other infections disorders (100) |
| Clindamycin | 1 (0.10) | Combination | 0 (0) |  |
|  |  |  |  |  |
| **Other antibacterial** | 67 (7.01) | Single antimicrobial | 51 (76.12) | Gastrointestinal disorders (50.74); Skin disorders (40.29); Other infections disorders (5.97); Other disorders (2.98) |
| Bacitracin | 29 (3.03) | Combination | 16 (23.88) |  |
| Fosfomycin | 1 (0.10) |  |  |  |
| Fusidic acid | 1 (0.10) |  |  |  |
| Metronidazole | 33 (3.45) |  |  |  |
| Mupirocine | 3 (0.31) |  |  |  |
